# Prevalence and contamination patterns of *Listeria monocytogenes* in *Pleurotus eryngii* (king oyster mushroom) production plants

**DOI:** 10.3389/fmicb.2023.1064575

**Published:** 2023-01-27

**Authors:** Jiang Xu, Shi Wu, Ming Liu, Zitian Xiao, Yangyang Peng, Huanqing He

**Affiliations:** ^1^Guangdong Key Laboratory for New Technology Research of Vegetables, Vegetable Research Institute, Guangdong Academy of Agricultural Sciences, Guangzhou, China; ^2^Guangdong Provincial Key Laboratory of Microbial Safety and Health, State Key Laboratory of Applied Microbiology Southern China, Institute of Microbiology, Guangdong Academy of Sciences, Guangzhou, China

**Keywords:** *Listeria monocytogenes*, *Pleurotus eryngii*, antimicrobial resistance, MLST, virulence genes

## Abstract

*Listeria monocytogenes* is a major foodborne pathogen that is well-known for its high mortality rate upon infection. In recent years, the edible mushroom has also been found to be an important source of *L. monocytogenes*, but the contamination sources in *Pleurotus eryngii* (the king oyster mushroom) were unclear. In this study, a total of 203 edible mushrooms and environmental samples from four *P. eryngii* production plants were obtained. As a result, 29 samples (14.3%) were positive for *L. monocytogenes*, including eight mushroom samples (13.3%, 8/60) and 21 associated environmental samples (14.7%, 21/143). The contamination of *L. monocytogenes* in plants A and B was more severe and was likely to originate from the mycelium stimulation machine. The isolates belonged to serogroups II.1 (4b-4d-4e), I.1 (1/2a-3a), and I.2 (1/2c-3c), and multilocus sequence typing (MLST) revealed that these *L. monocytogenes* strains belonged to five different sequence types (ST3, ST121, ST9, ST87, and ST224). The ST121 and ST3 isolates were only found in plants A and B, respectively. The isolates were carried by *hly* (29/29, 100%), *inlB* (23/29, 79.3%), *inlA* (29/29, 100%), *inlC* (29/29, 100%), *inlJ* (29/29, 100%), *actA* (19/29, 65.5%), *iap* (29/29, 100%), *plcA* (26/29, 100%), *plcB* (29/29, 100%), *prfA* (27/29, 93.1%), and *mpl* (29/29, 100%). Further study of *inlA* sequencing showed that 65.5% of strains (19/29) contained full-length InlA that was required for host cell invasion, whereas the mutation led to premature stop codons (PMSCs) at position 492 (type 6) on *inlA* alleles. All isolates in this survey were sensitive to gentamicin, kanamycin, sulbactam/ampicillin, trimethoprim-sulfamethoxazole, tetracycline, and doxycycline. The drug with the highest resistance is rifampicin (37.9%), followed by penicillin (24.1%) and ciprofloxacin (10.3%). Most multiply resistant strains are isolated from raw materials and equipment of the *P. eryngii* processing lines. Our study reflects the contamination patterns and potential risk of *L. monocytogenes* infection in *P. eryngii* production plants. The persistence of specific *L. monocytogenes* isolates (such as ST121 and ST3) may assist with contamination. In accordance with these results, the control of *L. monocytogenes* should focus on the environmental materials, especially in the mycelium stimulation stage. However, effective *Listeria* monitoring programs will allow for the improved development of *Listeria* control measures to minimize cross-contamination in the processing of *P. eryngii*.

## Introduction

*Listeria monocytogenes* is a major foodborne pathogen causing human listeriosis, a severe foodborne disease with high hospitalization rates (97.0%) and mortality rates (15.6%) (European Food Safety Authority [EFSA], and European Centre for Disease Prevention and Control [ECDC], 2019). Although *L. monocytogenes* accounted for 1% of foodborne disease outbreaks, it caused the highest percentage of foodborne disease-related deaths in the United States from 2009 to 2015 (Dewey-Mattia et al., 2018). The infection of *L. monocytogenes* involves the elderly, immunocompromised people, newborns, and pregnant women, which leads to sepsis, meningitis, encephalitis, abortion, and stillbirth (Lomonaco et al., 2009; Camargo et al., 2019). Its ability to survive and grow under a wide range of environmental conditions, including refrigeration temperatures, high salt concentrations, and low pH, makes it a potential hazard in foods (Ryser and Marth, 2007; Wu et al., 2016). L. monocytogenes causes listeriosis, with most cases caused by consuming contaminated food (Pérez-Rodríguez et al., 2017; El-Hajjaji et al., 2021). Meat, milk, and vegetables represent important sources of contamination, and *L. monocytogenes* can survive and multiply in raw products or ready-to-eat food made with such raw items, even if refrigerated (Filiousis et al., 2009).

Edible mushrooms, such as *Lentinula edodes*, *Auricularia auricula-judae*, *Pleurotus ostreatus*, *Flammulina velutipes*, *Pleurotus eryngii, Agaricus bisporus*, and *Auricularia polytricha*, are popular worldwide according to their taste and nutritional value. In 2020, the total output was 40.6 million tons in China, with an economic output of RMB ¥346.6 billion, accounting for ∼75% of global mushroom production. However, contaminations detected in food processing plants have led to rigid recalls of products and serious economic losses. In recent years, several recalls have occurred after detectable levels of *L. monocytogenes* were found on whole or sliced fresh mushrooms (Viswanath et al., 2013). More recently, *L. monocytogenes* was found contaminated in enoki mushrooms, which are responsible for 36 reported cases of listeriosis, including 31 hospitalizations and four deaths in the United States (Byun et al., 2022). Meanwhile, the occurrence of *L. monocytogenes* in edible mushroom products has been reported in several countries (Cordano and Jacquet, 2009; Venturini et al., 2011; Viswanath et al., 2013; Chen et al., 2018). However, it is necessary to understand how *L. monocytogenes* is entering mushroom production facilities through incoming raw materials and/or surviving and growing in farm and packing house environments.

Different molecular typing methods have been employed to assess the source of contamination and the routes of transmission of *L. monocytogenes*. The multilocus sequence typing (MLST) method is one of the most robust tools for investigating the global epidemiology of microbial populations (Sullivan et al., 2005). In addition, differentiation between virulent and hypo-virulent strains is significant for evaluating the potential implications of the presence of this microorganism for food safety and public health (Jensen et al., 2008). Among the 14 serotypes of *L. monocytogenes* identified, the results of multiplex polymerase chain reaction (PCR) indicated that these major serogroups could be separated into distinct groups: I.1 (1/2a-3a), I.2 (1/2c-3c), II.1 (4b-4d-4e), II.2 (1/2b-3b-7), and III (4a-4c) (Doumith et al., 2004). Serotypes 4b, 1/2a, and 1/2b are mostly associated with listeriosis in humans (Orsi et al., 2011; Poimenidou et al., 2018; Pérez-Baltar et al., 2021). Moreover, many virulence traits that participate in host invasion and cellular proliferation are mainly distributed in *Listeria* pathogenicity islands (LIPI), which have been identified depending on the pathogenicity of the strain and severity of the infection (Radoshevich and Cossart, 2018; Al et al., 2022). The *prfA*-regulated virulence gene cluster (*p*VGC), which consists of a monocistronic *hly* in the center of the locus, a lecithinase operon comprising *mpl, actA*, and *plcB* genes, and the *plcA-prfA* operon located upstream from *hly* and transcribed in the reverse direction are often referred to as *Listeria* pathogenicity island 1 (LIPI-1) (Vázquez-Boland et al., 2001a; Ward et al., 2004; Poimenidou et al., 2018). In addition, the internalins (InlA, InlB, InlC, and InlJ) play key roles in adhesion and invasion, which are essential for the pathogen to invade intestinal cells (Bonazzi et al., 2009). Furthermore, the determination of the genetic profile and antimicrobial susceptibility profile of *L. monocytogenes* isolates present in food sold in open markets may also help to trace future listeriosis outbreaks.

In a previous study, it was demonstrated that various retail edible mushroom samples such as *F. velutipes, P. eryngii, Volvariella volvacea, Hypsizygus marmoreus, Pleurotus geesteranus, P. ostreatus*, and *L. edodes* could both contaminate *L. monocytogenes* (Chen et al., 2018). Few other studies explored the contamination sources in *F. velutipes*, *H. marmoreus*, or other edible mushroom production facilities (Viswanath et al., 2013; Chen et al., 2014; Murugesan et al., 2015; Sun et al., 2021), but in *P. eryngii*, the situation is unclear. Considering that the processing of production for *P. eryngii* was similar to that of *F. velutipes* or *H. marmoreus*, this study was designed to investigate the prevalence of *L. monocytogenes* in four *P. eryngii* production plants and processing environments to explore the contamination sources and potential risk of the *L. monocytogenes* isolates in *P. eryngii* based on their genotypes determined by molecular serotyping and MLST, their antibiotic susceptibility, and their virulence gene profiles.

## Materials and methods

### Sampling and isolation of *L. monocytogenes*

A total of 203 samples were detected from edible mushrooms and environment samples in four king oyster mushroom (*P. eryngii*) production plants (A, B, C, and D) for contamination analyses. A sampling schematic of this study is shown in Table 1. Overall, each plant collected 47–52 samples, including 52 samples in plant A (Shaoguan, Guangdong), 52 samples in plant B (Yichun, Jiangxi), 47 samples in plant C (Suizhou, Hubei), and 52 samples in plant D (Chengdu, Sichuan). The samples were collected from different phases of production, including composting, bagging, the inoculation room (solid spawn, sterile compost, inoculating machinery surfaces, and inoculation room air), the breeding and mycelium stimulation machinery, the growing room (atomized water, shelf surfaces, mushrooms, and air), and the harvesting and refrigerating room (packaging machinery surfaces, scales, conveyor belts, and packaged mushrooms). The samples were placed in a cold box at a temperature of approximately 4°C, tightly sealed with sterile plastic wrap, transported to an accredited laboratory, and subjected to microbiological analysis within 24 h.

**TABLE 1:** Prevalence of *Listeria monocytogenes* in *Pleurotus eryngii* production plants.

Stages	Sampling details	Number of positive samples/total samples tested (%)				
		**Plant A**	**Plant B**	**Plant C**	**Plant D**	**Total**
Compost bagging stage	Unpasteurized compost	0/1 (0)	0/1 (0)	0/1 (0)	0/1 (0)	0/4 (0)
	Sterile compost	0/1 (0)	0/1 (0)	0/1 (0)	0/1 (0)	0/4 (0)
	Edible fungi compost bagging machine surfaces	0/1 (0)	0/1 (0)	1/1 (100)	0/1 (0)	1/4 (25.0)
	Transport machine surfaces	0/1 (0)	0/1 (0)	0/1 (0)	0/1 (0)	0/4 (0)
	Basket surfaces	0/1 (0)	0/1 (0)	0/1 (0)	0/1 (0)	0/4 (0)
	Shelf surfaces	0/1 (0)	0/1 (0)	0/1 (0)	0/1 (0)	0/4 (0)
Mycelium incubation stage	Solid spawn	0/5 (0)	0/5 (0)	0/4 (0)	0/5 (0)	0/19 (0)
	Basket surfaces	0/1 (0)	0/1 (0)	0/1 (0)	0/1 (0)	0/4 (0)
	Shelf surfaces	0/1 (0)	0/1 (0)	0/1 (0)	0/1 (0)	0/4 (0)
	Sir	0/1 (0)	0/1 (0)	0/1 (0)	0/1 (0)	0/4 (0)
Mycelium stimulation stage	Mycelium stimulation machine surfaces	3/5 (60.0)	4/5 (80.0)	0/3 (0)	0/5 (0)	7/18 (38.9)
	Floor	1/2 (50.0)	2/2 (100)	0/2 (0)	1/2 (50.0)	4/8 (50.0)
Growing control stage	Mushrooms	1/5 (20.0)	1/5 (20.0)	0/5 (0)	0/5 (0)	2/20 (10.0)
	Sir	0/1 (0)	0/1 (0)	0/1 (0)	0/1 (0)	0/4 (0)
	Water	0/1 (0)	0/1 (0)	0/1 (0)	0/1 (0)	0/4 (0)
	Shelf surfaces	0/2 (0)	1/2 (50.0)	0/2 (0)	0/2 (0)	1/8 (12.5)
	Drain	0/1 (0)	0/1 (0)	0/1 (0)	1/1 (100)	1/4 (25.0)
Harvesting stage	Mushroom	1/5 (20.0)	3/5 (60.0)	0/5 (0)	0/5 (0)	4/20 (20.0)
	Scales	0/1 (0)	0/1 (0)	0/1 (0)	0/1 (0)	0/4 (0)
	Knife	0/1 (100)	0/1 (0)	0/1 (0)	0/1 (0)	0/4 (0)
	Conveyor belts	1/1 (100)	0/1 (0)	0/1 (0)	0/1 (0)	1/4 (25.0)
	Drain	1/1 (100)	1/1 (100)	0/1 (0)	1/1 (100)	3/4 (75.0)
	Worker’s hands	1/1 (100)	0/1 (0)	0/1 (0)	0/1 (0)	1/4 (25.0)
Refrigerating stage	Mushroom product	1/5 (20.0)	1/5 (20.0)	0/4 (0)	0/5 (0)	2/19 (10.5)
	Outer packing surface	0/5 (0)	0/5 (0)	0/4 (0)	0/5 (0)	0/19 (0)
	Worker’s sole	1/1 (100)	0/1 (0)	0/1 (0)	1/1 (100)	2/4 (50.0)
Total		11/52 (21.2)	13/52 (25.0)	1/47 (2.1)	4/52 (7.7)	29/203 (14.3)

Strains were isolated in accordance with the following protocol based on GB/T4789.30-2010 of food microbiological examination of *L. monocytogenes* (National Food Safety Standards of China) with slight modification for qualitative detection. Approximately 25 g of sample was added to 225 ml of LB_1_ enrichment broth culture (Huankai, Guangzhou, China), which was incubated at 30°C for 24 h after homogenization in a stomacher bag. Then, 0.1 ml of LB_1_ enrichment broth culture was transferred to 10 ml of LB_2_ enrichment broth culture at 30°C for 24 h. A portion (10 μl) of LB_2_ enrichment broth culture was streaked onto *Listeria*-selective plates (CHROM-agar, Paris, France), which were incubated at 37°C for 24 h. Colonies with a blue halo were analyzed by Gram stain, catalase, and oxidase tests, and colonies were identified using a Micro ID *Listeria* identification system (Microgen, Camberley, UK).

### Molecular serotyping and multilocus sequence typing

Polymerase chain reaction assays were employed for molecular serogroups. A multiplex-PCR serogrouping of *L. monocytogenes* isolates was performed according to the instructions of Doumith et al. (2004) using five primers (*lmo*0737, *lmo*1118, ORF2819, ORF2110, and *prs*). This convenient method can characterize *L. monocytogenes* into five serogroups as follows: I.1 (1/2a-3a), I.2 (1/2c-3c), II.1 (1/2b-3b-7), II.2 (4b-4d-4e), and III (4a-4c).

The MLST scheme used to characterize *L. monocytogenes* isolates is based on the sequence analysis of the following seven housekeeping genes: *abcZ* (ABC transporter), *bglA* (beta-glucosidase), *cat* (catalase), *dapE* (succinyl-diaminopimelate desuccinylase), *dat* (D-amino acid aminotransferase), *ldh* (L-lactate dehydrogenase), and *lhkA* (histidine kinase) (Salcedo et al., 2003). The PCR amplification conditions were as follows: an initial cycle of 94°C for 4 min; 35 cycles of 94°C for 30 s, 52°C for 30 s (45°C for *bglA*), 72°C for 2 min, and a final extension at 72°C for 10 min. The DNA fragments were purified by using a PCR purification kit (Qiagen, Germany) and were sequenced in each direction with Big Dye fluorescent terminators on an ABI3730XL sequencer (Applied BioSystems).

For each MLST locus, an allele number was given to each distinct sequence variant, and a distinct sequence type (ST) number was attributed to each distinct combination of alleles at the seven genes. STs were determined by using the *Listeria monocytogenes* MLST database (Jolley et al., 2001). Sequence Type Analysis and Recombinational Tests software (S.T.A.R.T. ver. 2) was used to analyze the data of MLST.

### Determination of virulence-associated genes

Eleven virulence genes (*hly, inlB*, *inlA, inlC, inlJ, actA, iap*, *plcA, mpl, plcB*, and *prfA*) encoding the virulence proteins were chosen based on their importance in *L. monocytogenes* pathogenesis. PCR was performed to detect the presence of these virulence-related genes in the *L. monocytogenes* isolates. The relevant information is described in Supplementary Table 1. The amplicons were stained with GoldView, electrophoresed in 1.5% agarose at 120 V for 0.5 h, and visualized under a UV transilluminator gel imaging system (GE Healthcare, WI, USA). The images were saved as TIFF files for analysis.

The 2,400 bp long *inlA* gene was also sequenced in *L. monocytogenes* isolates. External primers were used for amplification covering the whole *inlA* ORF, and internal primers were used for sequencing (Supplementary Table 2). The *inlA* sequences were assembled using SeqMan (DNASTAR, Lasergene). Mutation types were determined according to the site of the mutation that leads to a premature stop codon (PMSC) in *inlA* (Nightingale et al., 2005) and by comparing the obtained *inlA* sequence data to that of the *L. monocytogenes* EGDe reference strain (Glaser et al., 2001).

### Antimicrobial susceptibility test

Antimicrobial susceptibility tests were performed using the standard disk diffusion Kirby–Bauer method on Mueller–Hinton agar, following the guidelines of the Clinical and Laboratory Standards Institute (Bauer et al., 1966; Granier et al., 2011; The Clinical and Laboratory Standards Institute [CLSI], 2018). Seventeen antibiotic disks (Oxoid, UK) were selected for the strains. These antibiotic disks were gentamicin (GEN, 10 μg), streptomycin (STR, 25 μg), kanamycin (KAN, 30 μg), chloramphenicol (CHL, 30 μg), rifampicin (RIF, 5 μg), cephalothin (CEF, 30 μg), levofloxacin (LEV, 5 μg), ciprofloxacin (CIP, 5 μg), vancomycin (VAN, 30 μg), clindamycin (CLI, 2 μg), erythromycin (ERY, 15 μg), ampicillin (AMP, 10 μg), penicillin (PEN, 10 U), sulbactam/ampicillin (SAM, 10/10 μg), trimethoprim-sulfamethoxazole (SXT, 23.75/1.25 μg), tetracycline (TET, 30 μg), and doxycycline (DOX, 30 μg). *Staphylococcus aureus* ATCC29213 and *Escherichia coli* ATCC25922 were used as quality control organisms (The Clinical and Laboratory Standards Institute [CLSI], 2018).

## Results

### Prevalence of *L. monocytogenes* in *P. eryngii* production plants

As shown in Table 1, it was found that 29 positive *L. monocytogenes* samples out of 203 detected samples showed a 14.3% prevalence in *P. eryngii* production plants in the present study. A total of 11 mushrooms and environment samples (21.2%, 11/52), 13 mushrooms and environment samples (25.0%, 13/52), 1 environment sample (2.1%, 1/47), and 4 environment samples (7.7%, 4/52) detected *L. monocytogenes* in plant A, plant B, plant C, and plant D, respectively. A total of 8 mushroom samples (13.3%, 8/60) were positive for *L. monocytogenes* in plants A and B, whereas 21 environmental samples (14.7%, 21/143) were positive in both four plants. The environment samples for detected *L. monocytogenes* involved compost bagging machine surfaces (25.0%, 1/4), mycelium stimulation machine surfaces (38.9%, 7/18), floor (50.0%, 4/8), drain (50.0%, 4/8), shelf surfaces (25.0%, 1/4), conveyor belts (25.0%, 1/4), worker’s hands (25.0%, 1/4), and worker’s sole (50.0%, 2/4). In different stages, the compost bagging stage and mycelium incubation stage were both free for *L. monocytogenes*, except one environment sample was positive in plant C. The two major stages of contamination were the mycelium stimulation stage and the harvesting stage. Meanwhile, the *L. monocytogenes* detected in mushroom samples focused on the growing control stage, the harvesting stage, and the refrigerating stage.

### Genotype diversity of *L. monocytogenes* isolates

A total of 29 *L. monocytogenes* isolates were collected from each positive sample. The isolates were carried out by molecular serotyping and MLST. All isolates were serotyped into three serogroups. Approximately 10 isolates (34.5%) belonged to serogroup I.1 (1/2a and 3a), four isolates (13.8%) belonged to serogroup I.2 (1/2c and 3c), and 15 isolates (51.7%) belong to serogroup II.1 (4b, 4d, and 4e), respectively. Serogroup II.2 (1/2b, 3b, and 7) and serogroup III (4a and 4c) did not exist in these isolates. Interestingly, all serogroup I.1 (1/2a and 3a) strains were isolated from plant A and most serogroup II.1 (4b, 4d, and 4e) from plant B, respectively.

**FIGURE 1:**
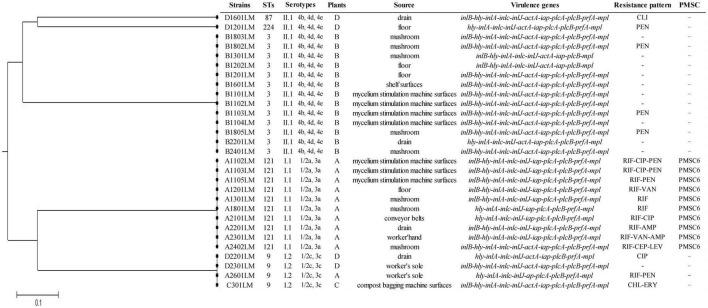
The unweighted pair group method with arithmetic mean (UPGMA) tree of the seven multilocus sequence typing loci of *L. monocytogenes* isolates from *Pleurotus eryngii* production plants. This tree was generated using the S.T.A.R.T (version 2).

By the MLST method, there were five different STs for all isolates. The most common allelic profile was ST3 (13/29, 44.8% of isolates), followed by ST121 (10/29, 34.5% of isolates), ST9 (4/29, 13.8% of isolates), ST87 (1/29, 3.5% of isolates), and ST224 (1/29, 3.5% of isolates). STs correlated well with molecular serotypes. Of the STs, ST121 belonged to serogroup I.1 (1/2a-3a), ST3, ST87, and ST224 belonged to serogroup II.1 (4b-4d-4e), and ST9 belonged to serogroup I.2 (1/2c-3c). The ST121 isolates were only found in plant A. Similarly, the ST3 isolates were only found in plant B. Except for one isolate (A2601LM) from plant A belonging to ST9, the other STs (ST9, ST224, and ST87) were respectively obtained from environmental samples in plant C and plant D. A phylogenetic tree based on the seven concatenated MLST sequences (Figure 1) shows the relatedness between the isolates.

### Virulence characterization of *L. monocytogenes* isolates

All 29 isolates in this study were examined for the presence of 11 virulence marker genes by PCR. As a result, all strains harbored *hly, inlA, inlC, inlJ, iap, mpl*, and *plcB* sequences, whereas 93.1% (27/29) of isolates harbored *prfA*, 89.7% (26/29) of isolates harbored *plcA*, 79.3% (23/29) of isolates harbored *inlB*, and 65.5% of isolates harbored *actA*. The full-length sequence of *inlA* was determined in all 29 isolates by DNA sequencing, and 65.5% (19/29) of isolates contained full-length InlA. It was found that 10 isolates harbor the same PMSC at position 492 (PMSC type 6) on *inlA* alleles. PMSC6 was detected in all tested ST121 strains.

### Antimicrobial susceptibility profiles

The antimicrobial susceptibility results of all isolates are shown in Table 2. Overall, the isolates in this survey were sensitive to gentamicin, kanamycin, sulbactam/ampicillin, trimethoprim-sulfamethoxazole, tetracycline, and doxycycline, except for two isolates with intermediate resistance to streptomycin. For 17 antibiotics, the highest resistance of *L. monocytogenes* isolates was rifampicin (11/29, 37.9%), followed by penicillin (7/29, 24.1%), ciprofloxacin (3/29, 10.3%), erythromycin (2/29, 6.9%), vancomycin (2/29, 6.9%), clindamycin (1/29, 3.5%), chloramphenicol (1/29, 3.5%), cephalothin (1/29, 3.5%), levofloxacin (1/29, 3.5%), and ampicillin (1/29, 3.5%). Ten isolates were susceptible to all 17 tested antibiotics, thirteen isolates were resistant to more than two antibiotics, and most of them were isolated from raw material and equipment of the edible mushroom processing lines.

**TABLE 2:** Results of antimicrobial susceptibility tests of *Listeria monocytogenes* isolates obtained from *Pleurotus eryngii* production plants.

Antimicrobial agent	Zone diameters (mm)			*L. monocytogenes* (*n* = 29)		
	**R**	**I**	**S**	**Number (%) of R**	**Number (%) of I**	**Number (%) of S**
Gentamicin (GEN)	≤12	13–14	≥15	0 (0)	0 (0)	29 (100)
Streptomycin (STR)	≤11	12–14	≥15	0 (0)	2 (6.9)	27 (93.1)
Kanamycin (KAN)	≤13	14–17	≥17	0 (0)	0 (0)	29 (100)
Chloramphenicol (CHL)	≤12	13–17	≥18	1 (3.5)	0 (0)	28 (96.6)
Rifampicin (RIF)	≤16	17–19	≥20	11 (37.9)	1 (3.5)	61 (55.2)
Cephalothin (CEF)	≤14	15–17	≥18	1 (3.5)	0 (0)	28 (96.6)
Levofloxacin (LEV)	≤15	16–18	≥19	1 (3.5)	0 (0)	28 (96.6)
Ciprofloxacin (CIP)	≤15	16–20	≥21	3 (10.3)	4 (13.8)	22 (75.9)
Vancomycin (VAN)	≤14	15–16	≥17	2 (6.9)	0 (0)	27 (93.1)
Clindamycin (CLI)	≤14	15–20	≥21	1 (3.5)	3 (10.3)	25 (86.2)
Erythromycin (ERY)	≤13	14–22	≥23	2 (6.9)	2 (6.9)	25 (86.2)
Ampicillin (AMP)	≤28	–	≥29	1 (3.5)	–	28 (96.6)
Penicillin (PEN)	≤28	–	≥29	7 (24.1)	–	22 (75.9)
Sulbactam/ampicillin (SAM)	≤11	12–14	≥15	0 (0)	0 (0)	29 (100)
Trimethoprim-sulfamethoxazole (SXT)	≤10	11–15	≥16	0 (0)	0 (0)	29 (100)
Tetracycline (TET)	≤14	15–18	≥19	0 (0)	0 (0)	29 (100)
Doxycycline (DOX)	≤12	13–15	≥16	0 (0)	0 (0)	29 (100)

R, resistant; I, intermediate resistance; S, susceptibility. The zone diameter was the standard from The Clinical and Laboratory Standards Institute [CLSI] (2018) of *Staphylococcus* spp., VA for *Enterococcus* spp.

## Discussion

In recent years, the contamination of *L. monocytogenes* has been found in edible mushrooms. Although few listeriosis outbreaks attributed to fresh mushrooms have been reported, recalls of mushroom products are common, caused by contamination with *L. monocytogenes* (Sun et al., 2021). *P. eryngii*, also known as the king oyster mushroom or cardoncello, is becoming increasingly popular with consumers in Europe, Asia, and North America. In recent times, it has become one of the most important commercial producers and exporters of edible mushrooms in China. However, the contamination source of *L. monocytogenes* in *P. eryngii* remains unclear. In this study, we investigated the prevalence of *L. monocytogenes* in four *P. eryngii* production plants from four cities and found that 14.3% of samples were positive for *L. monocytogenes*, including 13.3% of mushroom samples and 14.7% of environmental samples. The contamination is likely to originate from the mycelium stimulation machine, which coincides with previous research in the *F. velutipes* and *H. marmoreus* production facilities (Chen et al., 2014; Sun et al., 2021). In addition, the prevalence rate of *L. monocytogenes* in the *P. eryngii* production facility (14.3%) was higher than the *A. bisporus* production facility (1.6%) (Viswanath et al., 2013) but lower than the *H. marmoreus* production facility (27.0%) and the *F. velutipes* production facility (18.6%) (Chen et al., 2014). Compared with the mushroom samples (including the growing stage, harvesting stage, and products), our results were similar to those of *H. marmoreus* but lower than those of retail *F. velutipes* products (55.5%) (Chen et al., 2018). To our knowledge, the fruiting temperature of *F. velutipes* (6–12°C) is lower than that of *P. eryngii* (10–18°C) and *H. marmoreus* (12–16°C) in modern industrial cultivation of edible mushrooms. When *L. monocytogenes* was exposed to or persisted in the associated environment, its ability to proliferate at low temperatures may also play a key role in the persistence of this bacterium in harvesting and refrigerating stage product. Thus, the temperature accompanied by cross-contamination was probably the major reason for *L. monocytogenes* contamination in edible mushrooms.

As we know, different molecular typing methods have been employed to assess the source of contamination and the routes of transmission of *L. monocytogenes*. In this study, MLST was used and proved to be particularly suitable for the epidemiology investigations of food processing for *L. monocytogenes*. Overall, the source of *L. monocytogenes* contamination in the *P. eryngii* production plant was different and occurred inside the plant. Except for one isolate (A2601LM) belonging to ST9 from plant A, the isolates of *L. monocytogenes* focus on ST121 and ST3, which are found in plants A and B and originate from mycelium stimulation machines. The contamination was focused on *P. eryngii* production plants A and B, which are likely to originate from the mycelium stimulation stage. Meanwhile, in plants C and D, isolates from various STs (ST9, ST224, and ST87) were isolated from surfaces, floors, or drains, respectively. Thus, the *L. monocytogenes* contamination in the *P. eryngii* production plant could be caused by a wide variety of environments, but the contamination of the mycelium stimulation machine was the most important factor in this study. This is consistent with previous research in the *F. velutipes* and *H. marmoreus* production facility, which showed that the mycelium-scraping machine was the main transmission source in the production process (Chen et al., 2014; Sun et al., 2021).

Furthermore, the contamination of *L. monocytogenes* may also persist in *P. eryngii* production plants. Persistence has been defined as “repeated isolation on different dates of *L. monocytogenes* strains that are identified as identical subtypes (as determined by phenotypic or genotypic methods)” (Ferreira et al., 2014; Maury et al., 2016). According to previous studies, the *L. monocytogenes* ST121 strains, also found in this study, have previously been isolated from food and food processing facilities over several years in processing plants (Hein et al., 2011; Holch et al., 2013; Stoller et al., 2019). In Hein et al. (2011) found that the ST121 strains were isolated in Austria and Belgium from different ecological niches, including food and human cases, over several years. Similarly, Holch et al. (2013) reported that the ST121 strains isolated from four of eight different Danish fish processing industries were the persistent and dominant type in three plants over a period of 6 years, and Stoller et al. (2019) found that *L. monocytogenes* ST121 strains persisted in a meat processing facility over a 4-year period. According to the research of Harter et al. (2017), *L. monocytogenes* ST121 strains have the stress survival islet 2 (SSI-2) that is beneficial for survival under alkaline and oxidative stress conditions and is commonly encountered in food processing environments (Harter et al., 2017). All *L. monocytogenes* ST121 strains isolated from our study occurred PMSC mutations in *inlA*, which indicates that the ability of these persistent strains to attach to human host cells may decrease and also virulence attenuation (Van Stelten and Nightingale, 2008). Except for ST121, we also found ST3 isolates persisting in *P. eryngii* production plant B. These isolates belonged to serogroup II.1 (4b, 4d, and 4e), which was mostly related to food or humans with listeriosis (Sosnowski et al., 2019). According to the population structure analysis of *L. monocytogenes* isolates in Australia from 1931 to 2015, ST1 and ST3 were the most abundant strains (Jennison et al., 2017). In Poland, ST3 was the second most common type in *L. monocytogenes* isolates from various types of food of animal origin during 2013–2016 (Sosnowski et al., 2019). CC3 is the fourth most common clonal complex in Europe, is predominant in Australia, and was also detected in South and North America, Japan, and Oceania (Bespalova et al., 2021). Thus, the persistence of ST3 *L. monocytogenes* in the *P. eryngii* production plant deserves published attention. However, the reasons why ST3 *L. monocytogenes* persist in *P. eryngii* production plants remain to be further explored.

*Listeria monocytogenes* is classified into at least four evolutionary lineages (I, II, III, and IV) with different serotypes. Isolates of serotypes 1/2b, 3b, 3c, and 4b were assigned to lineage I, whereas isolates of serotypes 1/2a, 3a, and 1/2c were assigned to lineage II, and isolates of serotypes 4a and 4c were assigned to lineage III (Orsi et al., 2011). In this study, the *L. monocytogenes* isolates were serogroup II.1 (4b, 4d, and 4e), serogroup I.1 (1/2a and 3a), and serogroup I.2 (1/2c and 3c), which both belonged to lineage I and lineage II. As previously studied, most *L. monocytogenes* isolates belong to lineages I and II, which harbor the serotypes more commonly associated with human clinical cases, including serotype 1/2a (lineage II) and serotypes 1/2b and 4b (lineage I) (Orsi et al., 2011). Lineage I strains are mostly associated with the majority of human listeriosis outbreaks worldwide (Aureli et al., 2000; Jeffers et al., 2001; Gray et al., 2004), whereas lineage II strains are common in foods, seem to be widespread in natural and farm environments, and are also commonly isolated from animal listeriosis cases and sporadic human clinical cases (Lukinmaa et al., 2003; Parihar et al., 2008). Although *L. monocytogenes* has 14 serotypes that differ in virulence potential, serotypes 3a, 3b, 3c, 4a, 4c, 4e, and seven are very infrequent in food (Kathariou, 2002; Doumith et al., 2004; Yin et al., 2019). Thus, the dominant serotypes of *L. monocytogenes* isolated from the *P. eryngii* production facility should be 4b (15/29, 51.4%) and 1/2a (10/29, 34.5%), whereas 1/2c (4/29, 13.8%) is limited. However, the serotypes of *L. monocytogenes* isolates found in *P. eryngii* production plants have a close connection with human listeriosis outbreaks and should receive much attention.

In this study, the virulence genes, such as *hly, inlB*, *inlA, inlC, inlJ, actA, iap*, *plcA, mpl, plcB*, and *prfA*, were determined in *L. monocytogenes* isolates by PCR. These genes proved to play an essential role in the pathogenicity of L. monocytogenes. As a result, all isolates harbored at least nine of these genes. LIPI-1 (prfA, plcA, hly, mpl, actA, and plcB) genes were found in 55.2% (16/29) of the isolates, as well as the *iap* gene, which has an indirect role in their pathogenesis by codifying a product that is responsible for entering the host cell (Vázquez-Boland et al., 2001b; Iglesias et al., 2017). In addition, the *inlA* gene encodes a product that is responsible for the entry of the bacterium into the host cell, and the genes *inlB, inlC*, and *inlJ* are directly involved in the subsequent infection stages (Doumith et al., 2004; Iglesias et al., 2017). The *L. monocytogenes* strain isolated from the *P. eryngii* production plant contained *inlA, inlC*, and *inlJ*, whereas *inlB* was detected in 79.3% of isolates (23/29). However, the presence of either or both LIPI-1, *inlA, inlB, inlC*, and *inlJ* genes involved in *the L. monocytogenes* strain indicates that they had the ability to enter *via* the intraperitoneal route or through the use of contaminated food and cause infection. Furthermore, the mutations in *inlA* leading to a PMSC significantly reduce the invasion of the strain by human epithelial cells (Nightingale et al., 2005). However, only ten isolates from the sequencing of *inlA* genes had PMSC mutations, which means most of the strains (19/29, 65.5%) can produce full-length InlA, which is required for host cell invasion.

Regardless of the source, antimicrobial resistance within the isolates was significant. In this study, 17 clinically common antibiotics were tested for antimicrobial susceptibility in all *L. monocytogenes* strains. Compared with previous studies, the results of this study suggest that the overall incidence of antibiotic resistance in *L. monocytogenes* is relatively low (Pesavento et al., 2010; Rahimi et al., 2010; Camargo et al., 2015; Chen et al., 2018). Most *L. monocytogenes* isolates were sensitive to selected antibiotics. As the first-choice treatment for invasive listeriosis, only one isolate (3.5%, 1/29) and seven isolates (24.1%, 7/29) were resistant to ampicillin and penicillin. Besides, there was no resistance to the association of trimethoprim-sulfamethoxazole (an alternative treatment to invasive listeriosis). Erythromycin, a molecule recommended for the treatment of listeriosis in pregnant women (Charpentier and Courvalin, 1999), showed a mild level of resistance among the isolates (6.9% resistant and 6.9% intermediate resistance). *L. monocytogenes* is naturally susceptible to a wide range of antibiotics except for cephalosporin and fosfomycin (Hof et al., 1997). However, single- or multiple-resistant *L. monocytogenes* strains isolated from food, the environment, and clinics have been reported on a regular basis in recent years (Barbosa et al., 2013; Cufaoglu et al., 2021). Thus, as foodborne *L. monocytogenes* is the main cause of listeriosis, an antimicrobial susceptibility test for *L. monocytogenes* in food requires constant monitoring.

## Conclusion

In summary, the results of this study suggested that the contamination of *L. monocytogenes* in *P. eryngii* (king oyster mushroom) production plants is most likely caused by mycelium stimulation machines, with low temperatures during growth being the most likely cause. In addition, the persistence of specific *L. monocytogenes* isolates (such as ST121 and ST3) may assist with contamination. Most isolates belonged to molecular serotypes 4b-4d-4e and 1/2a-3a, which are associated with human listeriosis, suggesting that this pathogen may represent a potential danger to public health. Furthermore, the presence of either or both LIPI-1, *inlA, inlB, inlC*, and *inlJ* genes involved in the *L. monocytogenes* strain indicates that they had the ability to enter *via* the intraperitoneal route or using contaminated food and cause infection. Further study of *inlA* sequencing showed that most of the selected strains contained PMSC-lacking *inlA* gene sequences required for the encoding of the InlA factor and bacterial invasion of the host cell. Although the isolates we have found were not highly resistant, antimicrobial susceptibility tests for *L. monocytogenes* in food require constant monitoring. Our study reflects the potential risk of *L. monocytogenes* contamination in *P. eryngii* production plants. However, effective *Listeria* monitoring programs will allow for the improved development of *Listeria* control measures to minimize cross-contamination in processing *P. eryngii*.

## Data availability statement

The raw data supporting the conclusions of this article will be made available by the authors, without undue reservation.

## Author contributions

SW and JX conceived and designed the experiments and analyzed the data. JX and ML performed the experiments. HH and YP contributed to reagents, materials, and analysis tools. SW, JX, and ZX contributed to the writing of the manuscript. All authors contributed to the article and approved the submitted version.
