# Supplementary material for: Prevalence and contamination patterns of Listeria monocytogenes in Pleurotus eryngii (king oyster mushroom) production plants
Source: Front Microbiol. 2023 Jan 27;14:1064575. doi: 10.3389/fmicb.2023.1064575 (PMC9912299; doi:10.3389/fmicb.2023.1064575)
Supplement: Supplementary file 1 [file Data_Sheet_1.docx]

Supplementary Material

Prevalence and contamination patterns of *Listeria monocytogenes* in [*Pleurotus*](javascript:;)*[eryngii](javascript:;)* (king oyster mushroom) production plants

**Jiang Xu^1^, Shi Wu^2*^, Ming Liu^1^, Zitian Xiao^1^, Yangyang Peng^1^, Huanqing He^1^**

**Dr. Shi Wu:** [**wushiloveyou@126.com**](mailto:wushiloveyou@126.com)

# Supplementary Figures and Tables

## Supplementary Tables

**Supplementary Table 1.** Primer sequences used for *L. monocytogenes* isolates in this study

| **PCR test** | **Target gene** | **Ecoded protein/Function** | **Primer sequences (5’-3’)** | **Product sizes(bp)** | **References** |
| --- | --- | --- | --- | --- | --- |
| dPCR1 | *inlB* | Internalin B | F:GATATTGTGCCACTTTCAGGTT | 367 | Xu et al. (2009)^1^ |
|  |  |  | R: CCTCTTTCAGTGGTTGGGTT |  |  |
|  | *hly* | Listeriolysin O | F:GTTAATGAACCTACAAGACCTTCC | 707 |  |
|  |  |  | R: ACCGTTCTCCACCATTCCCA |  |  |
| mPCR2 | *inlA* | Internalin A | F: ACGAGTAACGGGACAAATGC | 800 | Liu et al. (2007)^2^ |
|  |  |  | R: CCCGACAGTGGTGCTAGATT |  |  |
|  | *inlC* | Internalin C | F: AATTCCCACAGGACACAACC | 517 |  |
|  |  |  | R: CGGGAATGCAATTTTTCACTA |  |  |
|  | *inlJ* | Internalin J | F: TGTAACCCCGCTTACACACAGTT | 238 |  |
|  |  |  | R: AGCGGCTTGGCAGTCTAATA |  |  |
| dPCR3 | *actA* | Actin–Polymerizing Protein | F: CGCCGCGGAAATTAAAAAAAGA | 839 | chen et al.(2014)^3^ |
|  |  |  | R: ACGAAGGAACCGGGCTGCTAG |  |  |
|  | *iap* | Invasion associated protein | F: ACAAGCTGCACCTGTTGCAG | 131 |  |
|  |  |  | R: TGACAGCGTGTGTAGTAGCA |  |  |
| PCR4 | *plcA* | Phospholipases C | F: CTGCTTGAGCGTTCATGTCTCATCCCCC | 1484 | chen et al.(2014)^3^ |
|  |  |  | R: CATGGGTTTCACTCTCCTTCTAC |  |  |
| PCR5 | *plcB* | Phospholipases C | F: ATGTGCTTGACCGCAAGTGT | 436 | chen et al.(2014)^3^ |
|  |  |  | R: CTTCTCGGTAATCAGCCACC |  |  |
| PCR6 | *prfA* | Transcriptional regulator | F: CTGTTGGAGCTCTTCTTGGTGAAGCAATCG | 1060 | chen et al.(2014)^3^ |
|  |  |  | R:AGCAACCTCGGTACCATATACTAACTC |  |  |
| PCR7 | *mpl* | zinc-metalloprotease | F: ATAGCTTTTCAGGCTCATTTCA | 1184 | chen et al.(2014)^3^ |
|  |  |  | R:A ATAGCTTTTCAGGCTCATTTCA |  |  |

**Reference:**

**1.** Xu XK, Wu QP, Zhang JM, Deng MQ and Zhou YH. Studies on specific detection of *Listeria monocytogenes* in foods by duplex PCR. Chinese Journal of Health Laboratory Technology. 2009;19: 1199-1221.

2. Liu D, Lawrence ML, Austin FW and Ainsworth AJ. A multiplex PCR for species-and virulence-specific determination of *Listeria monocytogenes*. J Microbiol Methods. 2007;71: 133-140.

3. Chen M, Wu Q, Zhang J, Yan Z and Wang J. Prevalence and characterization of *Listeria monocytogenes* isolated from retail-level ready-to-eat foods in South China. Food Control. 2014;38: 1-7.

**Supplementary Table 2.** Primers used for *inlA* PCR and sequencing

|  | Primer | Primer sequences (5’-3’) | Annealing temperature (°C) |
| --- | --- | --- | --- |
| Amplification primers | inlA F | CGGATGCAGGAGAAAATCC | 55 |
|  | inlA R | CTTTCACACTATCCTCTCC |  |
| Sequencing primers | CK_0814292_inlA.R | TAGCCAGAACACTAATATCC | - |
|  | CK_0814293_inlA.F1 | TGACGAATCTAACTGGTTTG | - |
|  | CK_0814294_inlA.F2 | AACAAGGTAAGTGACGTAAG | - |
|  | CK_0814295_inlA.F3 | CAAGAACCTACGGCACCAAC | - |
|  | HC_0817387_inlA.F3new^a^ | GTGGTGACAAGTGGGATTTC | - |

a D1201LM, D2201LM, D2301LM, B1102LM, B1201LM, B1202LM, B1301LM, B2401LM were used this primer for sequencing.
